# Effect of Combined Proteinuria and Increased Renal Resistive Index on Chronic Kidney Disease Progression: A Retrospective Longitudinal Study

**DOI:** 10.3390/jcm14010228

**Published:** 2025-01-03

**Authors:** Giulio Romano, Nicholas Fiorini, Martina Bertoni, Stefania Rondinella, Laura Di Pietra, Marco F. Cola, Paolo De Martin, Maurizio Tonizzo, Lorenzo Desinan, Benedetta Boari, Roberto Manfredini, GianLuca Colussi

**Affiliations:** 1 Nephrology Unit, Department of Medicine, University of Udine, 33100 Udine, Italy; giulio.romano@uniud.it (G.R.); nicholas.fiorini93@gmail.com (N.F.); bertoni.martina@spes.uniud.it (M.B.); lorenzo.desinan@uniud.it (L.D.); 2 Hypertension and Ultrasound Vascular Unit, Division of Internal Medicine, “Santa Maria degli Angeli” City Hospital of Pordenone, Azienda Sanitaria Friuli Occidentale, 33170 Pordenone, Italy; stefania.rondinella@asfo.sanita.fvg.it (S.R.); marco.cola@asfo.sanita.fvg.it (M.F.C.); paolo.demartin@asfo.sanita.fvg.it (P.D.M.); maurizio.tonizzo@asfo.sanita.fvg.it (M.T.); 3 Hypertension Unit, Division of Clinical Medicine, Department of Medical Sciences, University of Ferrara, 44124 Ferrara, Italy; benedetta.boari@unife.it (B.B.); roberto.manfredini@unife.it (R.M.)

**Keywords:** kidney function, interaction analysis, glomerular filtration rate, cardiovascular risk, atherosclerosis, urinary protein excretion

## Abstract

**Introduction:** An increased renal resistive index (RRI) and proteinuria can predict an estimated glomerular filtration rate (eGFR) decline in patients with chronic kidney disease (CKD) of various causes. This study hypothesized that the RRI and proteinuria interact to determine disease progression in patients with CKDs of unknown origin. **Patients and Methods**: One hundred and fifty six patients (age 76.0 ± 8.1 years, 63.5% males) were analyzed for anthropometric, kidney morphology, blood pressure, 24 h urinary protein excretion, and RRI. The CKD-EPI equation was used to calculate the eGFR at baseline and after a two-year follow-up. Patients with an elevated (≥0.80) or normal (<0.80) RRI and significant (≥150 mg/day) or physiological (<150 mg/day) proteinuria were evaluated for the likelihood of at least a 30% drop in the eGFR or the onset of end-stage kidney disease (endpoint). **Results**: Hypertension and diabetes were the predominant cardiovascular risk factors (90.4%). Fifty patients (32%) met the endpoint. Elevated RRIs (odds ratio, OR, 4.28; 95% confidence interval, CI, 1.82–10.6; *p* = 0.001) and significant proteinuria (OR 3.59, 95% CI 1.59–8.48, *p* = 0.003) were independent predictors of the endpoint in a multivariate logistic model. Patients with an elevated RRI and significant proteinuria were more likely to meet the endpoint (R1P1: 65.2%) compared to those with only proteinuria (R0P1: 39.5%, *p* = 0.043) or both normal factors (R0P0: 10.9%, *p* < 0.001) but not to those with only an elevated RRI (R1P0: 42.3%, *p* = 0.094). Continuous RRIs (partial correlation r = −0.245, *p* < 0.001) and 24 h urinary protein excretion (partial r = −0.226, *p* = 0.003) were inversely and independently correlated with eGFR% change. R1P1 showed a higher eGFR% reduction (−38.0% ± 20.4%) compared to R0P1 (−25.3% ± 19.0%, *p* = 0.043) and R0P0 (−8.8% ± 25.1%, *p* < 0.001) but not to R1P0 (−29.6% ± 21.0%, *p* = 0.192). **Conclusions**: An increased RRI and proteinuria were independent predictors of disease progression. When interaction was considered, the negative effect of an elevated RRI on CKD progression was evident in both proteinuric and non-proteinuric patients, whereas the negative effect of proteinuria on disease progression was only significant in patients with no elevated RRIs.

## 1. Introduction

Kidney function decline, cardiovascular events, and all-cause mortality are all linked to proteinuria in chronic kidney disease (CKD) [1]. Chronic proteinuria damages kidneys by increasing interstitial inflammation, oxidative stress, and glomerular filtration barrier degradation [2]. Thus, CKD diagnosis and treatment require early proteinuria detection and management. However, similar antiproteinuric strategies can reduce proteinuria in CKD patients with varying efficacy, and residual proteinuria remains a major risk factor for end-stage kidney disease (ESKD) [3]. Also, most CKD patients can develop ESKD without proteinuria [4]. Therefore, residual proteinuria or non-proteinuric patients who progress to ESKD may maintain risk through other pathways. Finding and targeting other kidney injury pathways and recognizing new markers may help slow CKD progression and reduce complications.

Proteinuria is diagnosed by quantifying 24 h urinary protein excretion. Most nephropathies, including those caused by hypertension and diabetes, are due to glomerular injury, and increased urinary protein excretion is the fundamental marker of kidney disease in these patients. Nephropathies with glomerular injury are closely related to urinary total protein or albumin excretion but neither measure is a better marker of CKD progression [5]. The 2021 Kidney Disease: Improving Global Outcomes (KDIGO) guidelines recommend assessing proteinuria, and the guidelines suggested conversion factors to compare different urinary protein measurements [6]. A protein-to-creatinine ratio of less than 15 mg/mmol indicates physiological-to-mild proteinuria, which is equivalent to 150 mg/day of total protein release in urine or 30 mg/day albumin excretion. Proteinuria above these cutoff values is significant and associated with poor renal outcomes, regardless of the glomerular filtration rate [6].

The risk of CKD progression is also associated with an increased renal resistive index (RRI), a measure of intrarenal blood flow by echo-color Doppler ultrasound [7]. In non-proteinuric CKD patients, an RRI equal to or higher than 0.80 was previously linked to a faster decline in kidney function and increased mortality [8]. CKD progression is also associated with an increased RRI in proteinuric patients [9,10], and the RRI is higher in proteinuric than in non-proteinuric patients [11]. We hypothesized that a high RRI combined with significant proteinuria would have a greater effect on CKD progression than either factor alone. This study investigated the effect of high RRIs, significant proteinuria, and their combination on kidney function decline or new onset ESKD after two years of follow-up in patients with CKD of unknown origin, which accounts for the vast majority of patients who attend our Nephrology Outpatient Clinic.

## 2. Patients and Methods

From June 2004 to January 2020, we screened non-dialyzing patients over the age of 18 of both sexes with KDIGO grade 2 to 4 CKD who were consecutively admitted to the Nephrology Outpatient Clinic of the Academic Hospital of Udine. Patients with 24 h urinary protein excretion in the nephrotic range (>3.5 g/day), who had acute or chronic glomerulonephritis, had started an angiotensin-converting enzyme inhibitor (ACE-i), angiotensin receptor blocker (ARB), or serum and glucose cotransporter-2 (SGLT-2) inhibitor, had cardiovascular events within 6 months prior, had other known kidney damage or renal artery stenosis as determined by a thorough diagnostic work-up, or had incomplete data were excluded. Renal function was assessed at baseline and after two years using the 2009 Chronic Kidney Disease Epidemiology (CKD-EPI) creatinine equation [12].

At the baseline visit, a blood sample was collected from the antecubital vein of each patient for laboratory analysis. Weight and height were recorded, and the body mass index (BMI) was calculated as weight in kilograms divided by squared height in meters. An automated oscillometric method (HEM-7143-E, Omron Healthcare Europe, Hoofddorp, The Netherlands) measured blood pressure in the dominant upper arm after 15 min of rest in the recumbent position. Hypertension is diagnosed by a history of high blood pressure, antihypertensive drug use, or repeated high in-office arterial blood pressure, with pulse pressure being the difference between systolic and diastolic blood pressure levels [13]. Diabetes was recorded when in at least two tests, fasting plasma glucose was ≥ 7.0 mmol/L (126 mg/dL), glycated hemoglobin was ≥ 48 mmol/mol (6.5%), or patients were taking antidiabetic medications [14]. The cardiovascular history was collected from anamnestic data or clinical records. Cardiovascular history included coronary or peripheral artery diseases, atrial fibrillation, heart failure, stroke, or transient ischemic attack (TIA) more than six months before.

Before starting ACE-i, ARB, or SGLT-2 inhibitors, 24 h urine samples were collected to assess proteinuria. The initial urine void was discharged the morning before the first visit, and other urine voids were collected in a box to calculate urine volume until the next day. Urine protein concentration was measured turbidimetrically. Kidney morphology and the RRI were assessed in each patient during the diagnostic work-up for CKD causes and to rule out renovascular disease using echo-color Doppler ultrasound (Affiniti 70 system, Philips, Eindhoven, the Netherlands, equipped with a 2.0–6.0 MHz Curved Array Transducer), as previously described [8]. Briefly, an interlobar artery and longitudinal kidney examination were performed on a dorsal decubitus patient at the first visit, averaging three Doppler waveforms at the upper, middle, and lower poles bilaterally. The bipolar length of the kidney was the largest distance between the upper and lower poles, and the cortical thickness was measured in the mid-kidney. The RRI was the difference between peak systolic and end-diastolic velocities divided by peak systolic velocity. All measurements were averaged over both kidneys to obtain the mean. In patients with suspected renal artery stenosis due to accelerated blood flow, a computed tomography or magnetic resonance imaging scan angiography was performed to rule out renovascular disease. Laboratory tests and imaging were performed by the Academic Hospital of Udine Division of Laboratory Medicine and Institute of Radiology, respectively. All exams were later reviewed by the same expert operators of the Hypertension and Ultrasound Vascular Unit, Division of Internal Medicine, City Hospital of Pordenone, to validate the measurements before including the patient in the study.

All patients signed at first visit an informed consent to use clinical data for research purposes. The electronic database, which contains anonymized clinical data, was password-protected and maintained on a server accessible only to the principal investigator and permitted coauthors. The Institutional Review Board of the University of Udine granted ethical approval for the current retrospective longitudinal analysis (protocol number 02/IRB-ROMANO_2019, approved on 13 February 2019).

### Statistical Analysis

Variables are reported as means ± standard deviations or medians (interquartile ranges) for normal or non-normal distributions, respectively. Normality was determined using a variable distribution histogram and the Shapiro–Wilk test. Student’s *t*-test for unpaired or paired data was used to compare means between or within subjects, respectively. The Tukey method was used to adjust for pairwise multiple means comparisons. Parametric analysis was performed on skewed variables after their log transformation. Fisher’s exact test was used to compare proportions. The study endpoint was established as either a drop in the eGFR of at least 30% or the onset of ESKD, characterized by an eGFR below 15 mL/min/1.73 m^2^, following a two-year follow-up [15]. We categorized proteinuria as <150 mg/day (physiological) or ≥150 mg/day (significant), and the RRI as <0.80 (normal) or ≥0.80 (elevated). For studying interaction, patients were classified into four distinct categories based on the dichotomized RRI and proteinuria variables, namely as a normal RRI with physiological proteinuria (R0P0), normal RRI with significant proteinuria (R0P1), elevated RRI with physiological proteinuria (R1P0), and elevated RRI with significant proteinuria (R1P1). The likelihood of predictors explaining the endpoint was expressed as an odds ratio (OR) with a 95% confidence interval (CI), calculated using logistic regression. To determine the independence of the RRI and proteinuria categories in determining the endpoint, we built a multivariate logistic model incorporating factors associated with the endpoint and elevated RRI or significant proteinuria, exhibiting *p* < 0.100. The Cochran–Armitage test for trends in proportion was used to test the hypothesis that the combination of RRI and proteinuria categories had an additive increasing effect on the likelihood of endpoint occurrence. In a secondary analysis, we used univariate and multivariate linear regression to investigate the determinants of eGFR% change with respect to baseline values, reporting the β coefficient of the regression line along with the *p*-value for a zero-slope hypothesis. Independent determinants were shown as partial regression plots after variable standardization. The plots illustrate the relationships between individual predictor variables and the response variable while controlling for the effects of other predictors. The slope of the regression lines represents the partial correlation coefficient (partial r) of each independent determinant considered in the multivariate model. The rate of the annual eGFR change for each category was evaluated with a linear mixed effects model and a post hoc comparison of estimated marginal means at baseline and follow-up.

In all statistical analyses, a *p*-value of less than 5% was considered significant for the rejection of the null hypothesis. The statistical analysis was conducted using R software (version 4.3.2, R Core Team, Vienna, Austria) [16]. The Hypertension and Ultrasound Vascular Unit at the City Hospital of Pordenone, along with the Hypertension Unit of the Division of Clinical Medicine at the University of Ferrara in Italy, performed the examination and interpretation of the data.

## 3. Results

### 3.1. General Study Group Characteristics

**Figure 1:**
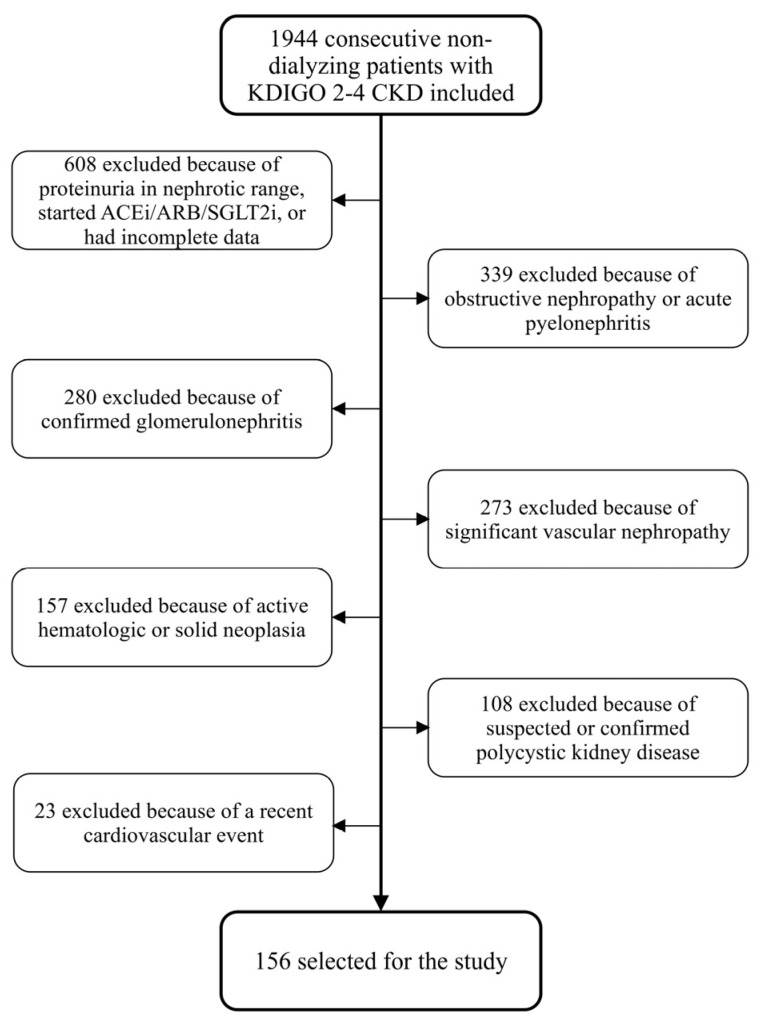
Flowchart summarizing the process of patient selection.

Of the 1944 patients screened, 156 met the study criteria and formed the study group (Figure 1).

General clinical characteristics of study patients are reported in Table 1. Most of the patients were older than 65 years (91%), and there was a higher prevalence of the male sex compared to the female sex, with a ratio of approximately 2:1. Hypertension and diabetes were the predominant cardiovascular risk factors, present alone or in combination in 90.4% of patients. Cardiovascular history consisted of 14.7% coronary artery disease, 14.7% heart failure, 14.7% stroke or TIA, 11.5% atrial fibrillation, and 7.7% peripheral artery disease. Statins and ezetimibe were the only lipid-lowering drugs used at baseline. All patients had on average kidney bipolar length and cortical thickness within the normal range. At baseline, 78.2% of patients had an eGFR below 60 mL/min/1.73 m^2^, increasing to 91.0% by the end of follow-up. ESKD was not present at baseline but developed in 12 patients (7.7%) by the conclusion of the follow-up period. Following a two-year follow-up, the eGFR% change with respect to the baseline value showed an overall decrease of −21.1% ± 24.6% across the entire cohort.

### 3.2. Clinical Characteristics of Study Patients According to the Endpoint

**Figure 2:**
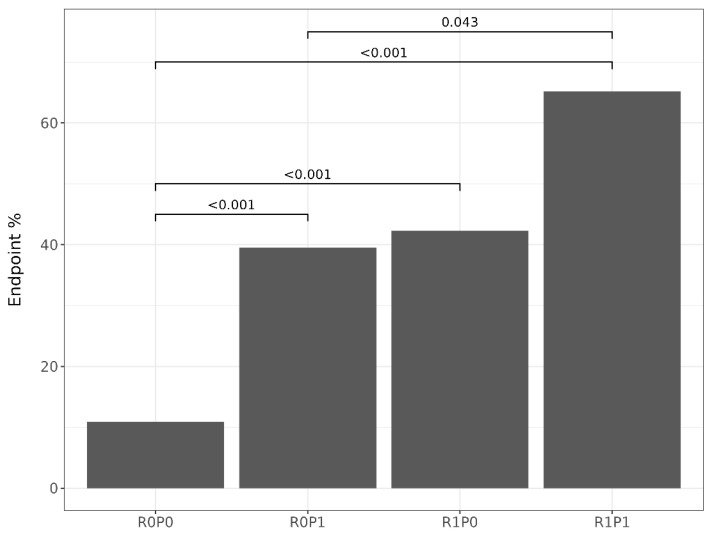
Prevalence of endpoint met in RRI x proteinuria categories with *p*-value of proportions pairwise comparison. See text for category’s definitions.

Fifty patients (32%) met the endpoint at two-year follow-up. Regarding patients who did not meet the endpoint, those who did were mostly of the male sex, had higher plasma creatinine levels, 24 h urinary protein excretion, and RRI values, and they had a higher prevalence of elevated RRIs and significant proteinuria, whereas they had lower baseline diastolic blood pressure and a lower eGFR at follow-up (Table 1). Patients in the R0P0 category were more frequent among those who did not meet the endpoint, while those in the R1P1 category were more frequent among those who met the endpoint (Table 1). The prevalence of the endpoint among patients classified into RRI x proteinuria categories is displayed in Figure 2. Patients classified as R0P0 had a lower endpoint prevalence (10.9%) than those in the R0P1 (39.5%), R1P0 (42.3%), and R1P1 (65.3%) categories, while the R1P1 category had a higher endpoint prevalence than the R0P1 (Figure 2). As the number of risk factors in the RRI × proteinuria categories increased (from R0P0 to R1P1), the Cochran–Armitage test showed a significant increasing trend in the prevalence of the endpoint (*p* < 0.001).

### 3.3. Baseline Predictors of the Endpoint

Baseline variables that predicted the endpoint by logistic regression analysis were male sex, lower diastolic blood pressure, higher plasma creatinine, an increased RRI, increased proteinuria, and each RRI × proteinuria category compared to R0P0 as a reference (Table 2). Baseline variables associated with an increased RRI were cardiovascular history and lower diastolic blood pressure; baseline variables associated with proteinuria were male sex and diabetes (Table 2). After having included all variables associated with the endpoint, elevated RRI, and significant proteinuria with a *p*-value lower than 0.100 in a multivariate logistic model, the analysis showed that an elevated RRI (OR 4.28, 95% CI 1.82–10.6, *p* = 0.001) and significant proteinuria (OR 3.59, 95% CI 1.59–8.48, *p* = 0.003) remained independent predictors of the endpoint occurrence (Table 3, Model 1). In a second multivariate logistic model, by considering the RRI x proteinuria categories instead of the dichotomized RRI and proteinuria variables, each category remained an independent predictor of the endpoint with a progressive increment of ORs with respect to the reference R0P0 category (Table 3, Model 2).

### 3.4. Determinants of eGFR Change with Respect to the Baseline Values

**Figure 3:**
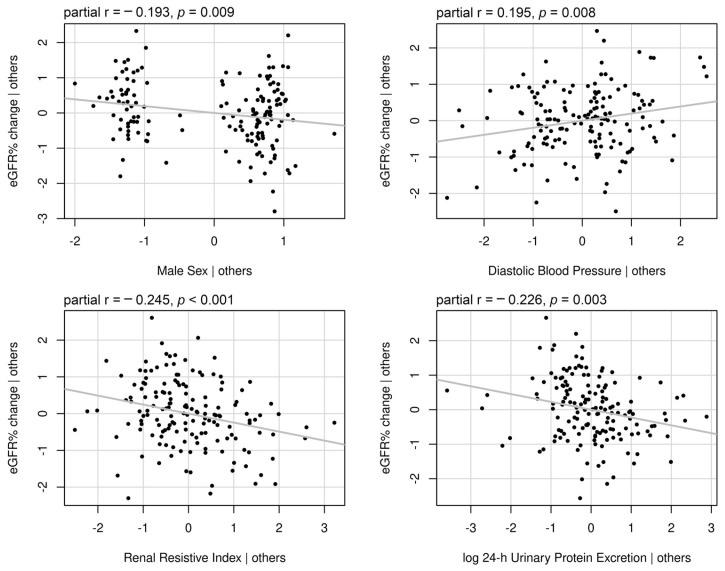
Partial regression plots of standardized variables independently associated with eGFR% change. The plots illustrate the relationships between individual predictor variables and the response variable while controlling for the effects of other predictors. The slope of the regression lines represents the partial correlation coefficient reported with the *p*-value on each plot.

**Figure 4:**
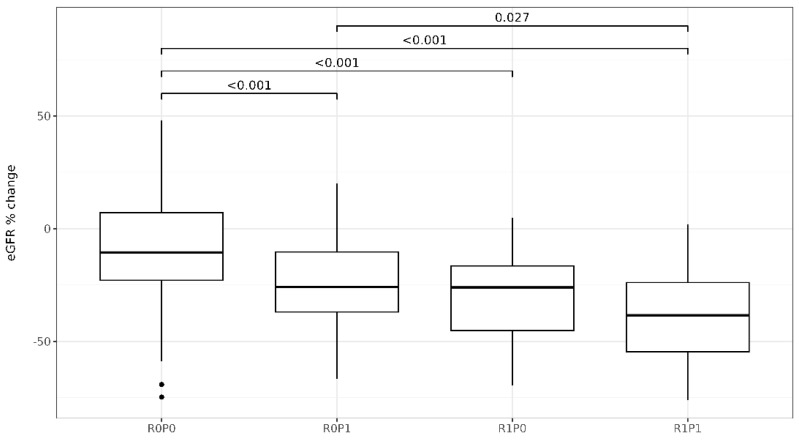
Boxplots of the eGFR% change compared to the baseline values across RRI x proteinuria categories analyzed via Student’s *t*-test for the hypothesis of decreasing trends with *p*-values corrected by the Tukey method. See text for the category’s definitions.

**Figure 5:**
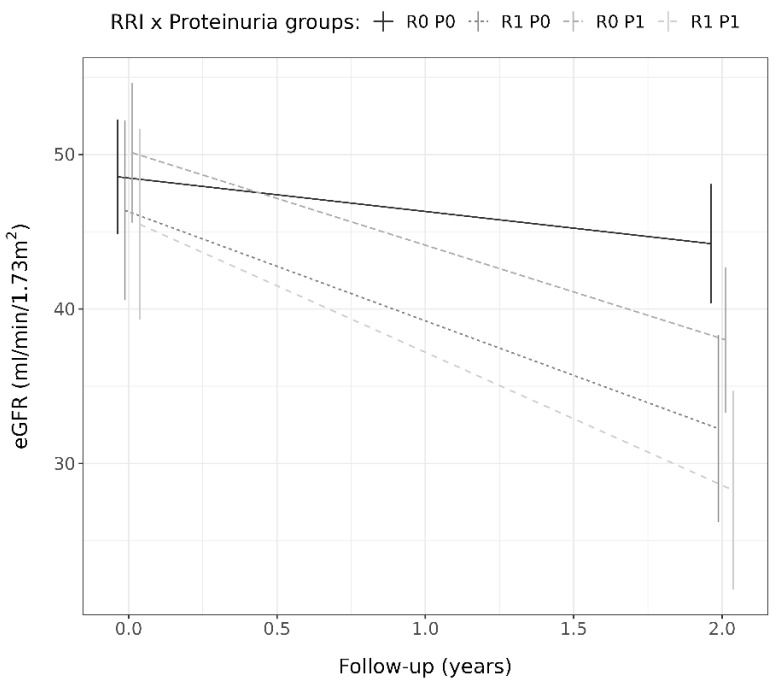
Estimated eGFR marginal means with 95% confidence intervals for each RRI x proteinuria category at baseline and follow-up, along with the corresponding annual change over time. See text for the category’s definitions.

Determinants of the eGFR% change with respect to the baseline values were male sex, diabetes, lower diastolic blood pressure, higher plasma creatinine levels, the RRI value, and log 24 h urinary protein excretion (Table 4). After including all determinants with a *p*-value lower than 0.100 in a multivariate linear model, only male sex, lower diastolic blood pressure, the RRI value, and log 24 h urinary protein excretion remained independently associated with the eGFR% change (Table 4). Figure 3 illustrates the partial regression plots of standardized independent determinants showing the correlation strength between each independent variable and eGFR% change. The RRI and log 24 h urinary protein excretion were the strongest correlated with eGFR% change. Figure 4 illustrates the change in eGFR% for patients classified into RRI x proteinuria categories. The eGFR% showed a greater reduction in patients classified within the R0P1 (−25.3% ± 19.2%, *p* < 0.001), R1P0 (−29.6% ± 21.0%, *p* < 0.001), and R1P1 (−38.0% ± 20.4%, *p* < 0.001) categories compared to those within the R0P0 (−8.8% ± 25.1%) and in those within the R1P1 category compared to those within R0P1 (*p* = 0.043). No differences in eGFR% change at follow-up were observed between the R0P1 and R1P0 categories (*p* = 0.400), as well as between R1P1 and R1P0 (*p* = 0.192, Figure 4). When RRI x proteinuria categories were considered as ordinate factors (R0P0 < R0P1 < R1P0 < R1P1), there was a significant linear trend (*p* < 0.001). Table 5 shows the estimated marginal means of absolute eGFR values with a 95% CI at baseline and follow-up, and Figure 5 illustrates the absolute eGFR change along time of follow-up. All categories showed a significant annual reduction in eGFR. No difference was observed between eGFR means at baseline (all *p* > 0.050), whereas the eGFR in R1P1 was lower than the eGFR in R0P1 (*p* = 0.034) and R0P0 (*p* < 0.001), and the eGFR in R1P0 was lower than R0P0 (*p* = 0.003) at follow-up (Table 5).

## 4. Discussion

The study showed that elevated RRIs (≥0.80) and significant proteinuria (24 h urinary protein excretion ≥ 150 mg/day) were independent predictors of the endpoint (30% eGFR reduction or new onset ESKD) after two years of follow-up. However, when examining the interaction between the RRI and proteinuria, the presence of either predictor at baseline increased the likelihood of the endpoint compared to neither, and the presence of both predicted a worse outcome than having only proteinuria or no predictors but not than having only a high RRI. These data suggest that an elevated RRI has the same negative impact on CKD progression in both non-proteinuric and proteinuric patients, whereas the negative impact of proteinuria on disease progression was only significant in patients who did not have an elevated RRI.

Our results are consistent with those reported by Radermacher et al. in hypertensive CKD patients without renal artery stenosis [9]. The authors found that an RRI > 0.80 and proteinuria >1 g/day were both independent predictors of CKD, defined as a 50% decrease in creatinine clearance or the need for dialysis. Furthermore, the authors reported that while the RRI and proteinuria were the only two independent predictors of kidney function decline, an increasing RRI was associated with a higher OR for the outcome than proteinuria. In comparison to the study by Radermacher et al., our study examined the interaction between the RRI and proteinuria, and the findings appear to support the hypothesis of the RRI playing a dominant role as a marker of CKD progression. However, the pathophysiological reasons for this observation remain unclear.

Pathological proteinuria in CKD patients is caused by single-nephron hyperfiltration and glomerular endothelial cell dysfunction [17]. Long-term oxidative stress, prothrombotic activity, and inflammation increase protein permeability in the glomerular filtration barrier [18,19], which causes glomerular and renal interstitial tissue damage, resulting in nephron sclerosis and kidney function decline [1]. Increased oxidative stress, prothrombotic factor activation, and inflammation have all been linked also to an increased RRI [20,21], thus suggesting a link between increased RRIs and proteinuria. However, while we know that an increased RRI is more common in proteinuric patients than non-proteinuric patients [11], that it can precede proteinuria development [22,23], and that it has been linked to early microalbuminuric stages of kidney dysfunction [24], there are no data on the development of increased RRIs in proteinuric patients, and speculations about the potential causal role of one factor versus the other factor can be limited.

An elevated RRI may indicate a precocious systemic hemodynamic alteration and microvascular damage, which characterize patients at elevated cardiovascular risk and initial kidney abnormalities [22,23,24,25]. Because increased RRIs can be found in CKD patients who have not yet developed proteinuria or will develop it later, it may be a more reliable indicator of kidney injury than proteinuria. However, when the RRI was added to a kidney failure prediction model that already included the eGFR, age, sex, and albumin-to-creatinine ratio, it improved model fitness but did not improve the discrimination of patients who would develop ESKD when compared to the original model [26]. This is an important point because increasing evidence suggests that the RRI is more closely associated with hemodynamic change, cardiovascular risk factors, and markers of systemic atherosclerosis than with functional or structural kidney abnormalities [22,23,27]. As a result, an increased RRI may be caused by systemic vascular dysfunction rather than a kidney-specific disease [26,28], and proteinuria can occur independently or as a result of systemic vascular damage that also affects kidney circulation.

As expected, after excluding causes of primary and secondary CKD with and without proteinuria, hypertension and diabetes remained the most common diseases associated with CKD in our cohort. In addition, our cohort consisted of elderly patients with a high prevalence of lipid-lowering therapy use and a significant history of cardiovascular disease, all factors that underlie vascular dysfunction and hemodynamic alterations. Lower diastolic blood pressure was found to predict both the endpoint and elevated RRI in this cohort. Low diastolic blood pressure may follow hemodynamic alterations seen in patients with increased RRIs and contribute to kidney damage because it is associated with aortic stiffness and peripheral flow pulsation, both of which are determinants of increased RRIs [29], proteinuria [30], and kidney dysfunction [31]. Low diastolic blood pressure can also impair renal tissue perfusion and accelerate eGFR decline, regardless of proteinuria [32,33]. Ponte et al. in a family-based population [34], Akaishi et al. in suspected CKD patients [35], and Kawai et al. in atherosclerotic patients [25] observed a similar independent and inverse relationship between the RRI and diastolic blood pressure. As a result, elevated cardiovascular risk and associated systemic hemodynamic dysfunction may have contributed to the observed dominant role of elevated RRIs as a predictor of CKD progression in our patients, implying that the RRI is an important marker of kidney disease progression in these high-atherosclerotic-risk patients, independent of proteinuria.

There were several limitations to this study that should be discussed. First, the retrospective design prevented us from determining which risk factors between an elevated RRI and significant proteinuria occurred first, limiting our ability to interpret our results. However, this is the first study to investigate the interaction between the RRI and proteinuria, and our findings should be regarded as preliminary in order to design future prospective studies. Second, because the study period lasted nearly two decades, RRI measurement methods changed over time, with multiple operators involved. Because Doppler measurements are operator-dependent, our results may have been limited by increased variability and wide confidence intervals. In addition, detailed Doppler ultrasound parameters other than the RRI, which was recorded in each screened patient, were not completely available, preventing further speculation about the characterization of renal artery Doppler parameters. Although this is an inherent concern in retrospective studies, we tried to minimize this problem by evaluating only completed Doppler data, and exams were later reviewed by the same expert operators to validate the measurements before including the patient in the study. Third, no information about disease-modifying drugs was provided during the follow-up. While the baseline evaluation and lab measurements were designed to exclude drugs that affect the renin–angiotensin–aldosterone system or the SGLT-2 receptor, the addition of these drugs during follow-up may have contributed to changes in kidney function decline. Probably, this has occurred primarily in proteinuric patients, where these medications are recommended by guidelines to reduce proteinuria and slow CKD progression [1]. Thus, the effect of proteinuria on CKD progression may be biased compared to that of increased RRIs. In addition, because the use of an SGLT-2 inhibitor to prevent CKD is much more recent than its use to treat diabetes, its use in our cohort of CKD patients is strongly associated with diabetes [36]. As a result, including a subanalysis on the effect of SGLT-2 inhibitors on CKD progression based on proteinuria and increased RRIs would be biased and ineffective. We believe that additional clinical trials should be conducted to determine the role of SGLT-2 inhibitors and other disease-modifying drugs in CKD prevention in patients with and without increased RRIs.

In conclusion, this study showed that the RRI and proteinuria were independent predictors of CKD progression. When the interaction was investigated, an elevated RRI was associated with higher CKD progression in both non-proteinuric and proteinuric patients, whereas proteinuria had a significant negative impact only in patients with no elevated RRI. Although these findings must be confirmed in prospective studies, the apparent dominant role of the RRI as a marker of CKD progression independent of proteinuria suggests that it should be evaluated in all patients with CKD of unknown origin.

## Figures and Tables

**Table 1:** General and by endpoint patients’ characteristics.

	Total	Endpoint Not Met	Endpoint Met	*p*
Count [n (%)]	156 (100)	106 (67.9)	50 (32.1)	-
Age (years)	76.0 ± 8.1	76.1 ± 7.6	75.9 ± 9.1	0.861
Male sex [n (%)]	99 (63.5)	56 (52.8)	43 (86)	<0.001
BMI (kg/m^2^)	30.6 ± 5.1	30.9 ± 5.0	29.9 ± 5.2	0.245
Cigarette smoking [n (%)]	30 (19.2)	21 (19.8)	9 (18)	0.832
Diabetes [n (%)]	51 (32.7)	30 (28.3)	21 (42.0)	0.102
Hypertension [n (%)]	138 (88.5)	95 (89.6)	43 (86.0)	0.593
Lipid-lowering therapy [n (%)]	82 (52.6)	55 (51.9)	27 (54.0)	0.864
Cardiovascular disease [n (%)]	69 (44.2)	45 (42.5)	24 (48.0)	0.632
SBP (mm Hg)	140 ± 17	141 ± 18	138 ± 16	0.303
DBP (mm Hg)	76 ± 9	77 ± 9	73 ± 9	0.024
PP (mm Hg)	64 ± 18	64 ± 18	64 ± 17	0.888
Plasma creatinine (mg/dL)	1.47 ± 0.38	1.42 ± 0.37	1.58 ± 0.38	0.011
Bipolar kidney length (cm)	10.35 ± 1.02	10.27 ± 1.02	10.53 ± 1.00	0.132
Kidney cortical thickness (cm)	1.37 ± 0.24	1.36 ± 0.23	1.40 ± 0.25	0.297
Renal resistive index	0.77 ± 0.06	0.76 ± 0.04	0.79 ± 0.07	0.001
RRI ≥ 0.80 [n (%)]	49 (31.4)	23 (21.7)	26 (52.0)	<0.001
24 h urinary protein excretion (mg/day)	122 [82–272]	120 [67–175]	233 [120–557]	<0.001
Proteinuria ≥ 150 mg/day [n (%)]	66 (42.3)	34 (32.1)	32 (64.0)	<0.001
Baseline eGFR (mL/min/1.73 m^2^)	48.2 ± 14.9	48.7 ± 14.8	47.0 ± 15.3	0.495
Follow-up eGFR (mL/min/1.73 m^2^)	38.2 ± 16.6	44.5 ± 14.7	24.7 ± 11.7	<0.001
New ESKD [n (%)]	12 (7.7)	0	12 (100)	<0.001
R0P0 [n (%)]	64 (41.0)	57 (53.8)	7 (14.0)	<0.001
R0P1 [n (%)]	43 (27.6)	26 (24.5)	17 (34.0)	0.297
R1P0 [n (%)]	26 (16.7)	15 (14.2)	11 (22.0)	0.319
R1P1 [n (%)]	23 (14.7)	8 (7.5)	15 (30.0)	0.001

BMI: body mass index; RRI: renal resistive index; SBP: systolic blood pressure; DBP: diastolic blood pressure; PP: pulse pressure; ESKD: end-stage kidney disease; eGFR: estimated glomerular filtration rate; R0P0: normal RRI with physiological proteinuria; R0P0: normal RRI with significant proteinuria; R1P0: high RRI with physiological proteinuria; R1P1: high RRI with significant proteinuria.

**Table 2:** Variables associated with the endpoint, elevated RRI, or significant proteinuria by univariate logistic regression.

	Endpoint Met		Elevated RRI		Significant Proteinuria	
	OR (95% CI)	*p*	OR (95% CI)	*p*	OR (95% CI)	*p*
Age (per 10 years)	0.96 (0.64–1.47)	0.860	1.15 (0.98–2.47)	0.073	0.89 (0.60–1.32)	0.565
Male sex (y/n)	5.49 (2.38–14.3)	<0.001	1.47 (0.72–3.07)	0.299	2.62 (1.32–5.37)	0.007
BMI (per 5 Kg/m^2^)	0.82 (0.57–1.14)	0.244	0.97 (0.69–1.35)	0.857	0.84 (0.61–1.15)	0.289
Smoking (y/n)	0.89 (0.36–2.06)	0.789	1.60 (0.69–3.64)	0.262	0.89 (0.39–1.99)	0.776
Diabetes (y/n)	1.83 (0.91–3.71)	0.091	1.30 (0.63–2.64)	0.467	2.42 (1.23–4.85)	0.011
Hypertension (y/n)	0.71 (0.26–2.05)	0.510	2.50 (0.78–11.2)	0.164	1.17 (0.44–3.36)	0.755
Lipid-lowering therapy (y/n)	1.09 (0.56–2.15)	0.805	1.31 (0.66–2.61)	0.439	1.76 (0.93–3.37)	0.086
Cardiovascular disease (y/n)	1.25 (0.64–2.46)	0.515	2.75 (1.38–5.60)	0.004	0.98 (0.52–1.86)	0.950
SBP (per 10 mm Hg)	0.90 (0.73–1.09)	0.302	0.92 (0.74–1.11)	0.391	1.16 (0.96–1.40)	0.128
DBP (per 10 mm Hg)	0.65 (0.44–0.94)	0.027	0.63 (0.42–0.92)	0.021	1.04 (0.73–1.48)	0.836
PP (per 10 mm Hg)	1.01 (0.84–1.23)	0.887	1.04 (0.86–1.25)	0.713	1.14 (0.95–1.37)	0.160
Plasma creatinine (per 1 mg/dL)	3.20 (1.30–8.22)	0.013	2.16 (0.89–5.39)	0.091	1.47 (0.64–3.43)	0.369
Kidney bipolar length (per 1 cm)	1.29 (0.93–1.79)	0.132	1.08 (0.77–1.50)	0.666	1.27 (0.93–1.74)	0.142
Kidney cortical thickness (per 1 cm)	2.12 (0.52–8.83)	0.296	0.96 (0.23–3.96)	0.960	1.04 (0.27–3.93)	0.955
RRI ≥ 0.80 (y/n)	3.91 (1.91–8.14)	<0.001	-	-	1.32 (0.66–2.61)	0.429
Proteinuria ≥ 150 mg/day (y/n)	3.77 (1.88–7.76)	<0.001	1.32 (0.66–2.61)	0.429	-	-
Baseline eGFR (per 10 mL/min/1.73 m^2^)	0.92 (0.73–1.16)	0.493	0.86 (0.67–1.08)	0.213	1.03 (0.83–1.27)	0.814
RRI × proteinuria categories:						
R0P0 (Reference) R0P1 R1P0 R1P1	1.00 5.32 (2.04–15.2) 5.97 (2.02–18.9) 15.3 (5.00–52.2)	- 0.001 0.002 <0.001				

**Table 3:** Multivariate logistic regression results of the endpoint predictors.

	Model 1		Model 2	
	OR (95% CI)	*p*	OR (95% CI)	*p*
Age (every 10 years)	0.91 (0.54–1.55)	0.717	0.91 (0.54–1.55)	0.782
Male sex (y/n)	4.77 (1.71–15.2)	0.005	4.78 (1.71–15.2)	0.005
Diabetes (y/n)	1.32 (0.54–3.18)	0.540	1.29 (0.53–3.12)	0.566
Lipid-lowering therapy. (y/n)	0.79 (0.34–1.79)	0.567	0.75 (0.32–1.73)	0.503
Cardiovascular disease (y/n)	0.66 (0.27–1.55)	0.350	0.66 (0.27–1.54)	0.341
DBP (every 10 mm Hg increase)	0.60 (0.37–0.96)	0.037	0.60 (0.37–0.96)	0.035
Plasma creatinine (per 1 mg/dL)	1.77 (0.53–5.95)	0.351	1.76 (0.53–5.94)	0.354
RRI ≥ 0.80 (y/n)	4.28 (1.82–10.6)	0.001	-	-
Proteinuria ≥ 150 mg/day (y/n)	3.59 (1.59–8.48)	0.003	-	-
RRI x proteinuria categories:				
R0P0 (Reference) R0P1 R1P0 R1P1			1.00 4.93 (1.70–15.8) 6.32 (1.90–22.8) 14.7 (4.28–57.3)	- 0.005 0.003 <0.001

**Table 4:** Univariate linear regression β coefficient and multivariate adjusted β coefficient of baseline variables associated with eGFR% change.

	Univariate Analysis		Multivariate Analysis	
Variable	β Coefficient	*p*	β Coefficient	*p*
Age (years)	0.050	0.838	-	-
Male sex (0/1)	−13.93	<0.001	−8.99	0.029
BMI (Kg/m^2^)	0.469	0.232	-	-
Cigarette smoking (0/1)	2.869	0.568	-	-
Diabetes (0/1)	−8.254	0.049	−2.24	0.575
Hypertension (0/1)	−4.367	0.481	-	-
Lipid-lowering therapy (0/1)	−2.525	0.524	-	-
Cardiovascular disease (0/1)	−3.991	0.317	-	-
SBP (mm Hg)	0.118	0.300	-	-
DBP (mm Hg)	0.643	0.003	0.50	0.012
PP (mm Hg)	−0.056	0.614	-	-
Plasma creatinine (mg/dL)	−12.56	0.015	−2.55	0.623
Kidney bipolar length (cm)	−2.534	0.192	-	-
Kidney cortical thickness (cm)	−7.785	0.346	-	-
RRI	−142.23	<0.001	−104.74	0.002
24 h urinary protein [log(mg/day)]	−6.772	<0.001	−4.90	0.005
Baseline eGFR (mL/min/1.73 m^2^)	0.080	0.548	-	-

**Table 5:** Estimated eGFR marginal means and annual rate of eGFR reduction (with 95% CI) of patients across RRI × proteinuria categories.

RRI x Proteinuria Category	eGFR mL/min/1.73 m^2^ at Baseline	eGFR mL/min/1.73 m^2^ at Follow-Up	Rate eGFR Reduction mL/min/1.73 m^2^/Year	*p*
R0P0	48.5 (44.8–52.3) ^a^	44.2 (40.4–48.1) ^b^	−2.2 (0.7–3.6)	0.005
R0P1	50.1 (45.6–54.6) ^a^	38.0 (33.3–42.7) ^c^	−6.0 (4.6–7.5)	<0.001
R1P0	46.4 (40.6–52.2) ^a^	32.3 (26.2–38.3) ^c,d^	−7.1 (4.8–9.3)	<0.001
R1P1	45.5 (39.3–51.7) ^a^	28.3 (21.8–34.7) ^d^	−8.6 (6.5–10.7)	<0.001

Similar letters in column or row analysis indicate no statistical difference. The reported *p*-value is only related to the annual rate of eGFR reduction.

## Data Availability

The datasets generated and analyzed during the current study are available from the corresponding author upon reasonable request and after authorization by the Administrative Department of the University of Udine.
